# Exposure of cattle to tick-borne encephalitis virus in the historical endemic zone in north-eastern France

**DOI:** 10.1186/s12917-024-04079-8

**Published:** 2024-05-25

**Authors:** Laure Mathews-Martin, Gaëlle Gonzalez, Nolwenn M. Dheilly, Rayane Amaral-Moraes, Marine Dumarest, Teheipuaura Helle, Camille Migne, Christophe Caillot, Sandrine A. Lacour, Sylvie Pérelle, Cécile Beck, Raphaëlle Metras, Laure Bournez

**Affiliations:** 1 ANSES, Nancy Laboratory for Rabies and Widlife, Malzéville, F-54220 France; 2 VetAgro Sup, ENSV-FVI, Marcy-L’Étoile, F-69280 France; 3 ANSES, INRAE, ENVA, UMR Virology, ANSES Animal Health Laboratory, Maisons-Alfort, F-94700 France; 4 ANSES, Laboratory for Food Safety, UVE, Maisons-Alfort, F-94700 France; 5 Sorbonne Université, INSERM, Pierre Louis Institute of Epidemiology and Public Health (IPLESP, UMRS, 1136), Paris, F-75012 France

**Keywords:** Tick-borne encephalitis virus, cELISA, Cattle, Seroprevalence

## Abstract

**Background:**

Tick-borne encephalitis (TBE) is a severe human neuroinfection caused by TBE virus (TBEV). TBEV is transmitted by tick bites and by the consumption of unpasteurized dairy products from infected asymptomatic ruminants. In France, several food-borne transmission events have been reported since 2020, raising the question of the level of exposure of domestic ungulates to TBEV. In this study, our objectives were (i) to estimate TBEV seroprevalence and quantify antibodies titres in cattle in the historical endemic area of TBEV in France using the micro virus neutralisation test (MNT) and (ii) to compare the performance of two veterinary cELISA kits with MNT for detecting anti-TBEV antibodies in cattle in various epidemiological contexts. A total of 344 cattle sera from four grid cells of 100 km² in Alsace-Lorraine (endemic region) and 84 from western France, assumed to be TBEV-free, were investigated.

**Results:**

In Alsace-Lorraine, cattle were exposed to the virus with an overall estimated seroprevalence of 57.6% (95% CI: 52.1–62.8%, *n* = 344), varying locally from 29.9% (95% CI: 21.0–40.0%) to 92.1% (95% CI: 84.5–96.8%). Seroprevalence did not increase with age, with one- to three-year-old cattle being as highly exposed as older ones, suggesting a short-life duration of antibodies. The proportion of sera with MNT titres lower than 1:40 per grid cell decreased with increased seroprevalence. Both cELISA kits showed high specificity (> 90%) and low sensitivity (less than 78.1%) compared with MNT. Sensitivity was lower for sera with neutralising antibodies titres below 1:40, suggesting that sensitivity of these tests varied with local virus circulation intensity.

**Conclusions:**

Our results highlight that cattle were highly exposed to TBEV. Screening strategy and serological tests should be carefully chosen according to the purpose of the serological study and with regard to the limitations of each method.

## Introduction

Tick-borne encephalitis (TBE) is the most common arbovirosis in Europe with more than 3,000 reported human cases per year [1]. TBE results from infection with tick-borne encephalitis virus (TBEV), which belongs to a group of viruses (TBEV serocomplex) from the *Flavivirus* genus (family *Flaviviridae*). This serocomplex includes louping-ill virus (LIV) and other viruses causing LI-like diseases in small ruminants [2]. In Western Europe, only the European subtype (TBEV-Eu) naturally circulates between the tick *Ixodes ricinus* (reservoir species and vector) and forest rodents (known competent hosts). TBE causes severe human neuroinfection with a fatal outcome in 0.5 to 2% of cases [3]. TBEV is mainly transmitted to humans via bites from infected ticks. However, food-borne transmission has also been regularly reported in Eastern and Central Europe after consumption of unpasteurized dairy products from infected domestic ungulates [3]. Food-borne outbreaks mainly occur in clusters [4–12]. Most cases are associated with raw dairy products from goats [13]. The food-borne route represents approximately 1% of all TBE human cases reported since 2012 to the European Centre for Disease Prevention and Control (ECDC), but the total number of food-borne cases is probably underestimated.

In France, since the first detection of TBE in 1968, its incidence has remained low with 10 to 30 autochthonous cases per year [14, 15]. A slight increase in the number of reported cases has been observed in recent years, with the detection of new foci [16]. However, the distribution of the virus is not well known in France, and cases can be misdiagnosed. The first food-borne outbreak in France occurred in 2020 in the department of Ain in the Auvergne-Rhône-Alpes region (AURA). Forty-three people fell ill with TBE with neurological disorders after having eaten goat dairy products from a single producer [17]. After this event, TBE became a notifiable disease in May 2021 [18]. Since then, food-borne transmission has been highly suspected for 6.5% of the new notified autochthonous cases. These events raise the concern of TBE food-borne outbreaks in France, Europe’s leading producer and consumer of raw goat cheese.

To better assess the risk of food-borne TBE cases in France, there is a crucial need for more data on the extent of viral circulation and on the exposure of domestic ungulates to the virus in the country. Moreover, testing sera from small ruminants or cattle for the presence of TBEV antibodies can be useful to evaluate viral circulation and identify new natural foci in a country before diagnosing the first human cases [19–31]. Indeed, domestic ruminants are hosts for various *I. ricinus* life stages. After infection, they usually remain asymptomatic, developing low-level TBEV viraemia. They can excrete the virus into milk for up to 23 days [4], and develop an antibody response [4, 32]. However, much remains unknown about immunity and longevity of antibodies in ruminants. One study reported that antibodies can persist for three to six years in goats and sheep immunized multiple times with a human-adapted vaccine [24], but another study suggested that they persist at low levels for less than one or two years in naturally infected animals [21]. However, a weak antibody response was also observed in several individuals in the latter study, with no information on potential re-infections in these animals, leaving the possibility of a weak, intermittent and long-lasting antibody response. To date, no data are available on exposure of domestic ruminants to TBEV in France, except from the serological survey conducted on the herd of goats incriminated in the 2020 food-borne outbreak and on the surrounding farms [17]. TBEV exposure in this herd was relatively high with 25% seropositive goats (*n* = 56). In France, small ruminants are not evenly distributed across the country, unlike cattle which are present almost everywhere. In addition, blood samples are collected every year on suckling cows for brucellosis and infectious bovine rhinotracheitis (IBR) surveillance, whereas samples for small ruminants are only taken every five years. Thus, cattle can be a promising sentinel species to use for assessing TBEV circulation in France and exposure of domestic ruminants to TBEV.

Detection of TBEV antibodies in animal sera is mainly based on a micro virus neutralisation test (MNT) and commercial or in-house enzyme-linked immunoabsorbent assays (ELISAs) [33]. Because cross-reactions are frequent among flaviviruses, MNT is the gold standard test to identify the virus by detecting specific neutralising antibodies against viruses in serum samples. Nevertheless, MNT cannot neither distinguish which virus of the TBEV serocomplex is responsible for the seroconversion, nor detecting non-neutralising antibodies. Furthermore, MNT requires appropriate biosafety conditions in confinement laboratories, is time-consuming and difficult to implement for the large numbers of samples routinely used in epidemiological serological surveys. Competitive ELISAs (cELISAs) have been developed to detect specific antibody fractions (immunoglobulin G [IgG] and/or immunoglobulin M [IgM]) in animal serum samples. These tests are rapid and easy to perform, but sensitivity and specificity can vary substantially in commercially available assays, especially in the absence of species-specific thresholds for detecting positive samples [33–35]. They may detect specific non-neutralising antibodies undetectable by MNT which could explain positivity in ELISA and not in MNT. ID Screen® West Nile Competition (ID Vet, Montpellier, France) is designed to detect West Nile Virus (WNV), but the high cross-reactivity observed makes this test suitable for the detection of a broad range of flavivirus antibodies such as TBEV or Usutu virus (USUV) [36]. Immunozym FSME IgG all species (Progen, GmbH, Germany) is another commercial multispecies cELISA veterinary kit, but specifically developed to detect TBEV. Both of them are frequently used for screening in large TBEV serological surveys conducted on wild and domestic fauna. These commercial kits have been compared for the detection of TBEV antibodies in various species, but not in cattle and not always by adapting the threshold values to the targeted species [35, 37–39]. Recently, one study, although based on a very limited number of TBEV positive samples, has shown that their capacity to detect positive samples was especially low when seroneutralising antibody titres were low [37]. If seroneutralising antibody titres vary with circulation intensity or age of animals - supposing that reinfection and thus antibody titres increase with virus circulation intensity or age - sensitivity of the tests may therefore vary with epidemiological contexts. This deserves to be further studied.

The first goal of this study was to assess seroprevalence and quantify the titres of anti-TBEV neutralising antibodies in cattle in an endemic area in France (Alsace-Lorraine, north-eastern France). We studied seroprevalence and titre variation according to age class to gain insight into the intensity of virus circulation in recent years (young animals), and to obtain clues as to the longevity of antibodies in cattle (particularly in older cattle). We hypothesized that a long-lived duration of TBEV antibodies and multiple re-infection events lead to an increase of TBEV seroprevalence and MNT titre with age. Moreover, higher local virus prevalence would be associated with higher risk of re-infections and higher antibodies titres. Secondly, we compared the analytical performance of the two cELISA kits mentioned above with that of MNT to detect anti-TBEV IgG antibodies, using the recommended cutoff values or those optimized for cattle serum samples. In particular, we studied the sensitivity of cELISA kits according to MNT titre and assessed how it varied with local virus circulation intensity and cattle age. Lastly, we assessed the influence of the chosen screening method on the estimation of seroprevalence in serological surveys.

## Results

### Epidemiological characteristics of TBEV circulation in the endemic area

**Fig. 1:**
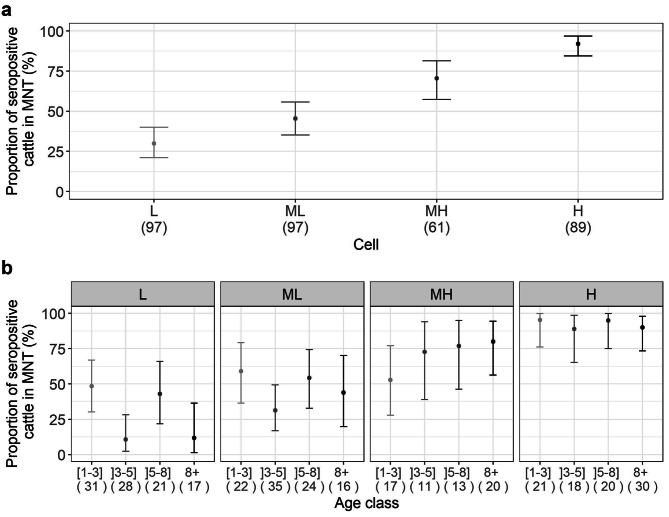
(**a**) Proportion of seropositive cattle for each grid cell using MNT test (L: low, ML: medium low, MH: medium high, H: high seroprevalence levels) and (**b**) per age class and per cell, the numbers within brackets represent the sample size

The study was conducted in four zones of 100 km², which were designated as follows: cell “L”, cell “ML”, cell “MH” and cell ”H”. The overall TBEV seroprevalence detected using MNT was high, with 57.6% (95% CI: 52.1–62.8%) of positive cattle (198/344), varying from 29.9% (95% CI: 21.0–40.0%) in L cell (*n* = 97) to 92.1% (95% CI: 84.5-96.8%) in H cell (*n* = 89) (Fig. 1a). Seroprevalence did not increase with age. In all cells, the [1–3] year-old category (*n* = 91) showed high seroprevalence, greater than 45%, revealing recent and intense viral circulation (Fig. 1b). According to generalized linear mixed model (GLMM) results, the probability of an individual being TBEV seropositive did not vary significantly according to age category.

**Fig. 2:**
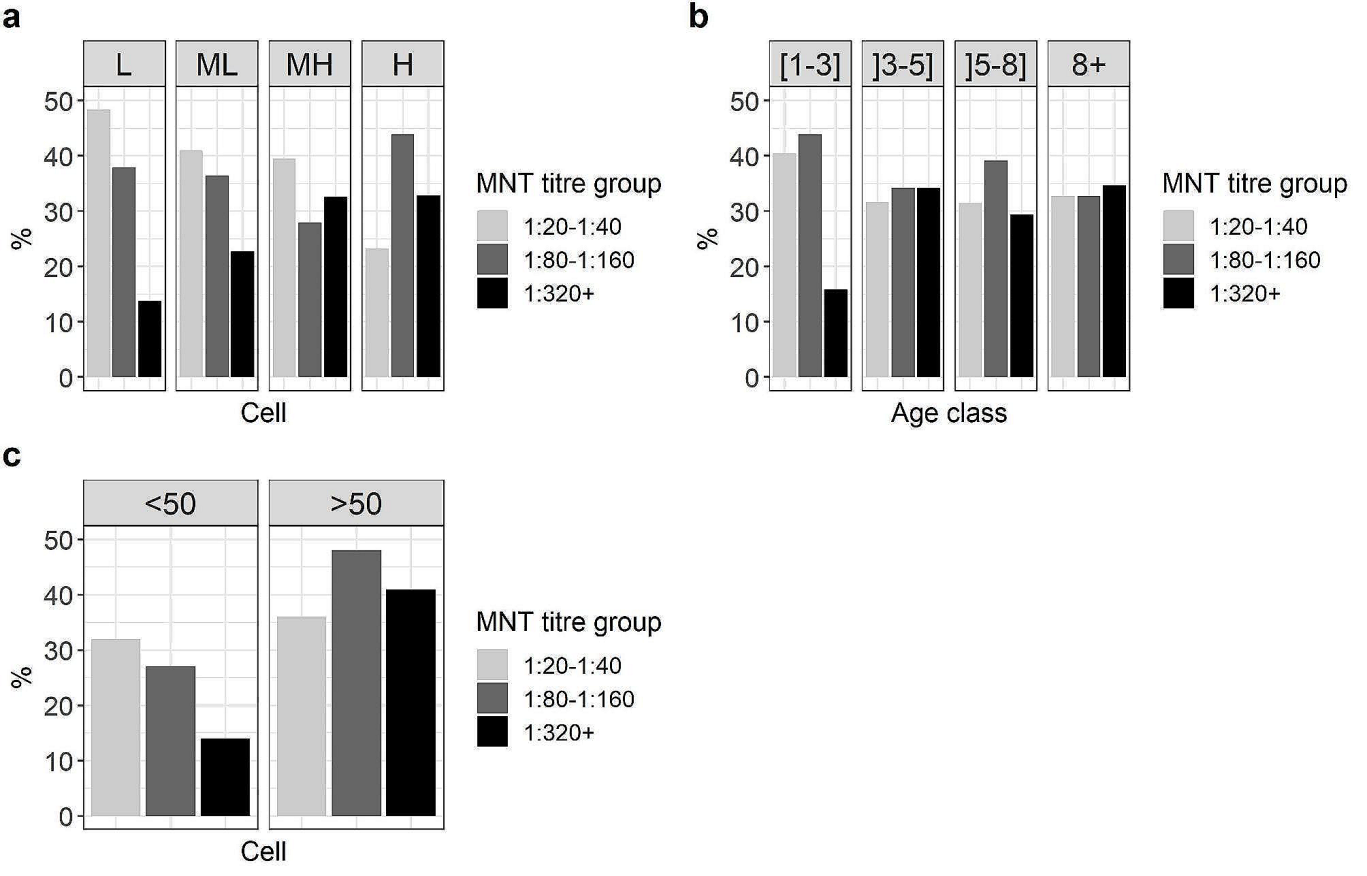
(**a**) Proportion of sera with low titres (1:20 − 1:40), medium titres (1:80 − 1:160) and high titres (≥ 1:320) of neutralising antibodies among positive sera per cell (L: low, ML: medium low, MH: medium high, H: high seroprevalence levels), (**b**) per age class, and (**c**) per cell with a seroprevalence below 50% (< 50) and with a seroprevalence above 50% (> 50)

The proportion of low (1:20 − 1:40) and high (≥ 1:320) titres of MNT antibodies among positive samples varied with the level of seroprevalence in the cell and age class (Fig. 2a and b). The proportion of high titres (≥ 1:320) in H cell (seroprevalence > 90%) was significantly higher than in L cell (seroprevalence < 30%) (Fisher test, *p*-value = 0.05), and inversely for the proportion of low titres (χ²=5.3, *p*-value = 0.02). In L cell, animals with low MNT titres were 3.5 times more frequent than those with high titres. The proportion of individuals in each titre group varied only among the [1–3] year-old cattle, with three times as many cattle with high titres than low titres. Furthermore, the proportion of cattle with titres above 1:320 was significantly lower in the [1–3] class than in the other age classes (χ²=6.1, *p-*value = 0.01). However, the results of the multinomial logistic models indicate that only the intensity of virus circulation was significantly associated with the MNT titre group, whereas cells were grouped by seroprevalence into two categories “<50%” and “>50%” (likelihood ratio test, *p*-value = 0.08) (Fig. 2c).

### Performance of cELISA kits compared with MNT

**Fig. 3:**
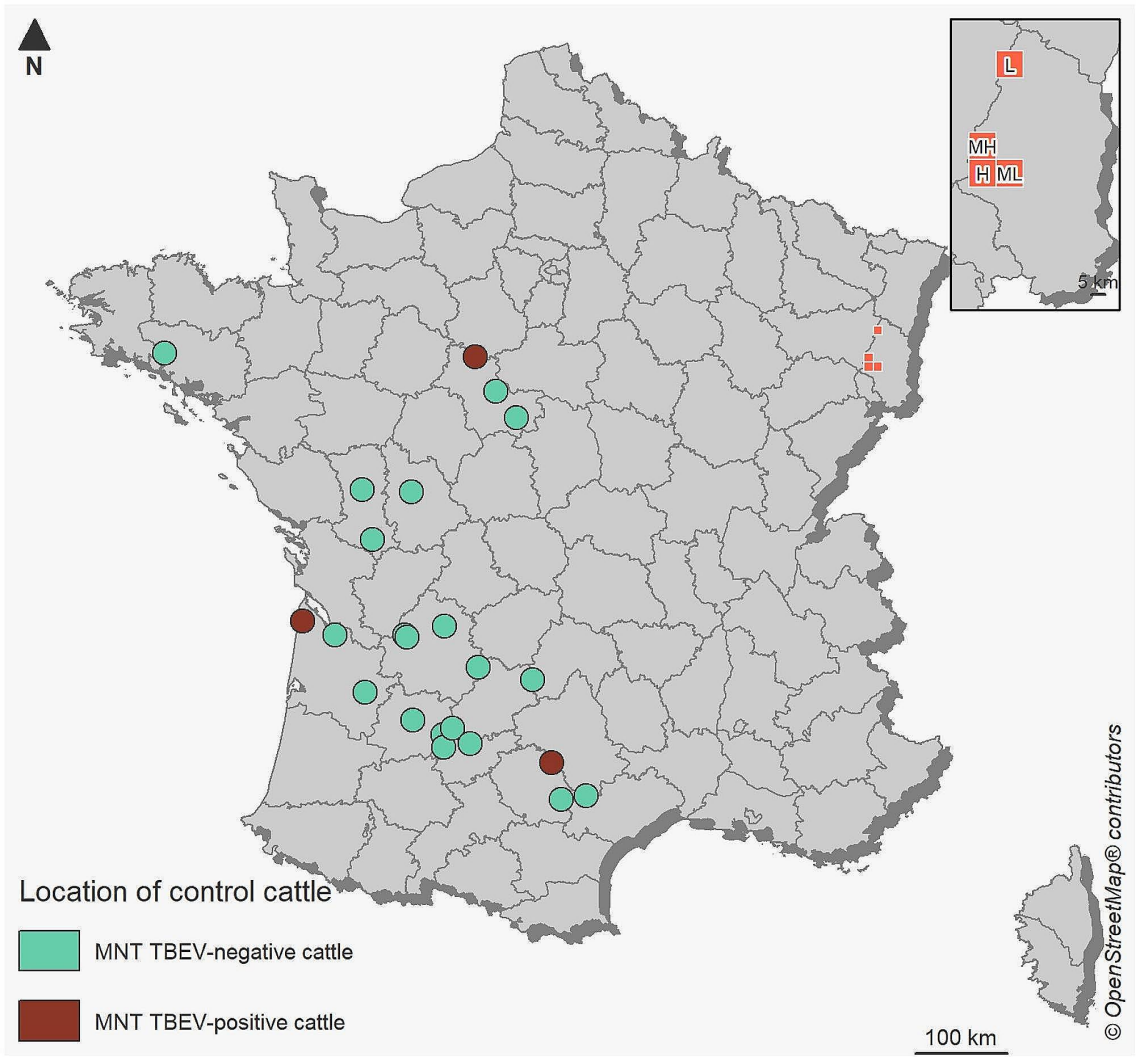
Study area. Location of the 84 animals sampled in presumed TBEV-free areas (control animals) including the three MNT-positive cattle and location of the four 10 × 10 km² cells in the TBEV endemic area in north-eastern France, in which 344 cattle were sampled, with L: low circulation, ML: medium-low circulation, MH: medium-high circulation and H: high circulation

The performance of cELISA kits was tested on 201 positive and 227 negative serum samples in MNT, including 84 sera in presumed TBEV-free area in France. MNT-positive sera included 198 serum samples from the endemic area and — surprisingly — 3 samples from western France presumed TBEV-free: a one- and a two-year-old female born and raised in the same farm in Eure-et-Loir and Gironde, respectively, with a titre of 1:20, and a 10-year-old female in Tarn, raised on the same farm since 2013 with a titre of 1:40 (Fig. 3). These three cattle samples were all tested negative for USUV in MNT. For the following analyses, these sera were considered TBEV-positive.

**Table 1:** Relative sensitivity (Se), relative specificity (Sp), kappa coefficient (ƙ) and area under the curve (AUC) of the two cELISA (ID Screen® cELISA, Immunozym cELISA) kits compared with the reference MNT test on 428 cattle samples

	ID Screen® cELISA					Immunozym cELISA			
Cutoff	50		86			63		62	
	positive	negative	positive	negative		positive	negative	positive	negative
MNT positive	31	170	130	71		151	50	156	45
MNT negative	2	225	56	171		22	205	22	205
Se (%)	15.4		64.5			75.1		78.1	
Sp (%)	99.1		75.3			90.3		90.3	
ƙ	0.11		0.40			0.68		0.68	
AUC (%)	78					89			

Cutoffs 50 and 63: doubtful thresholds recommended by the manufacturers; cutoffs 86 and 62: optimal positive thresholds weighting sensitivity and specificity

Considering the doubtful thresholds recommended by the manufacturers, specificity was high (> 90%) for both tests, but associated with moderate sensitivity for Immunozym cELISA (75.1%) and poor sensitivity for ID Screen® cELISA (15.4%), thus resulting in a high number of false-negative sera. We then calculated optimal positive thresholds (weighting sensitivity and specificity equally) for the detection of cattle antibodies. For ID Screen® cELISA, optimal positive cutoff was 86 (S/N) instead of the recommended one of 40 (40 ≥ S/N for positive, 40 < S/N ≤ 50 for doubtful) and 62 VIEU/ml instead of 126 (63 < concentration ≤ 126 for doubtful, 126 ≤ concentration for positive) for Immunozym cELISA. For this cELISA, the new estimated threshold corresponded to the doubtful manufacturer’s threshold and therefore the performance of the test was not improved. In contrast, the sensitivity of ID Screen® cELISA improved, reaching 64.5% to the detriment of specificity (75.3%). ID Screen® cELISA was nevertheless less effective than Immunozym cELISA for all the parameters evaluated although Immunozym cELISA stayed moderately sensitive (78.1%) with a degree of agreement with MNT being only substantial (ƙ = 0.68) (Table 1). Specificity values were similar when using only sera from the presumed TBEV-free area. All samples tested with ID Screen® cELISA that presented positive and doubtful results according to manufacturer threshold (39/344) were positive for TBEV and negative for USUV using MNT, except one serum sample that was MNT-negative for both viruses.

**Fig. 4:**
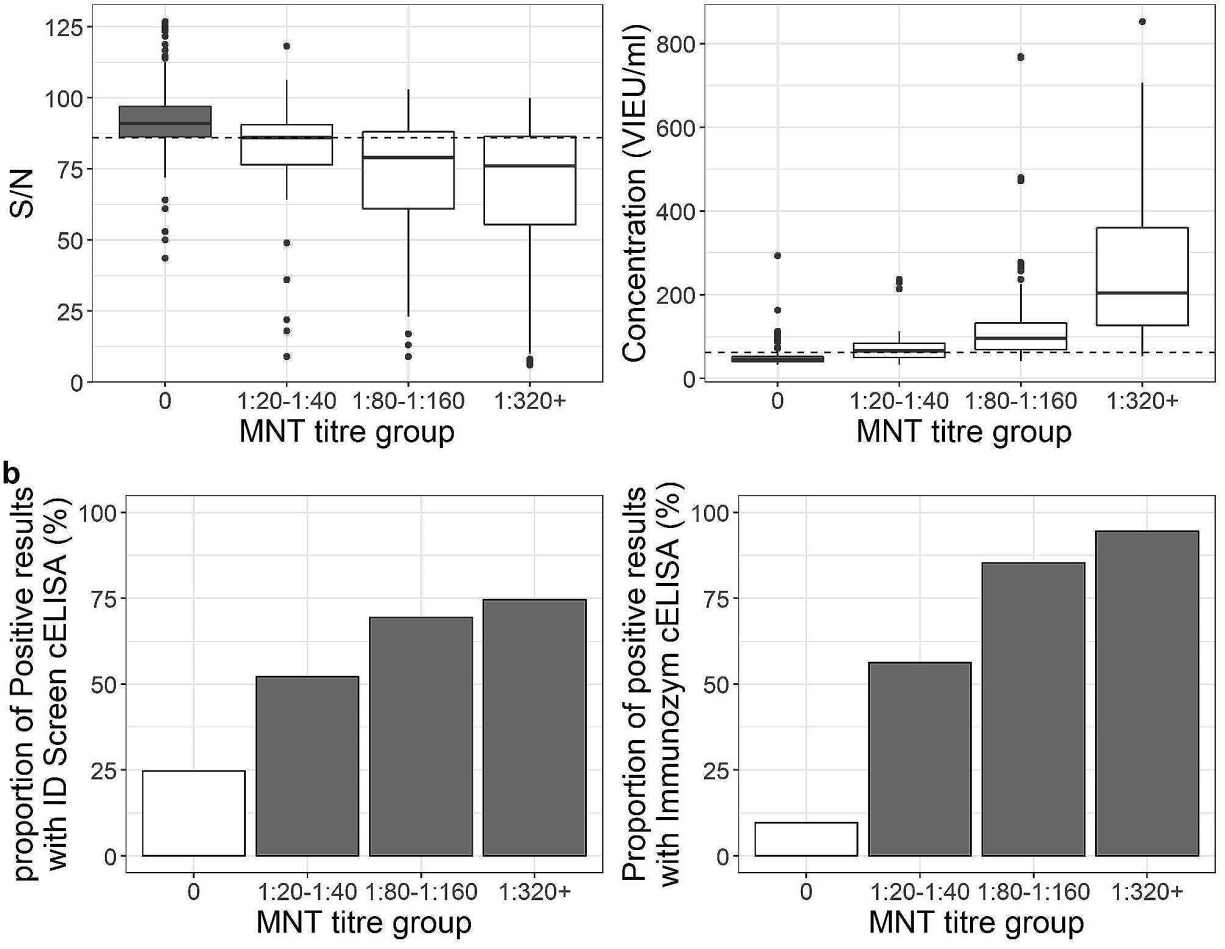
(**a**) Relationship between titre group in MNT and cELISA results and (**b**) the proportion of positive cELISA results per titre group (optimized thresholds 86 S/N and 62 VIEU/ml)

Using our threshold optimizing sensitivity and specificity, 186 sera were positive with ID Screen® cELISA and 178 with Immunozym cELISA. Results from the two cELISA kits were consistent for 312 sera, with 124 positive and 188 negative results for both kits. These 124 positive sera were all confirmed positive in the TBEV MNT, except 9 samples. Among the 188 negative results for both kits, 30 tested positive in MNT: one with a titre of 1:320, three with a titre of 1:160 and 26 with titres less than 1:80. The detection of TBEV-specific antibodies by both cELISA kits was correlated to the amount of neutralising antibodies in samples. Concentration in VIEU/ml obtained with Immunozym cELISA was more closely correlated to antibody titre in MNT than S/N results obtained with ID Screen® cELISA (Spearman correlation coefficients, 0.73 and 0.47, respectively, *p*-values < 0.05). Both cELISAs were less effective in detecting TBEV-positive cattle when neutralising antibody concentrations were low (≤ 1:40), with less than 60% of serum samples with a titre of 1:20 − 1:40 MNT-positive. ID Screen® cELISA’s ability to detect positive sera stayed relatively low even for high titres (less than 75% of positive results), whereas it reached 95% with Immunozym cELISA when the titre measured using MNT was at least 1:320 (Fig. 4a and b).

### Variation of estimated seroprevalence according to chosen screening strategy

**Fig. 5:**
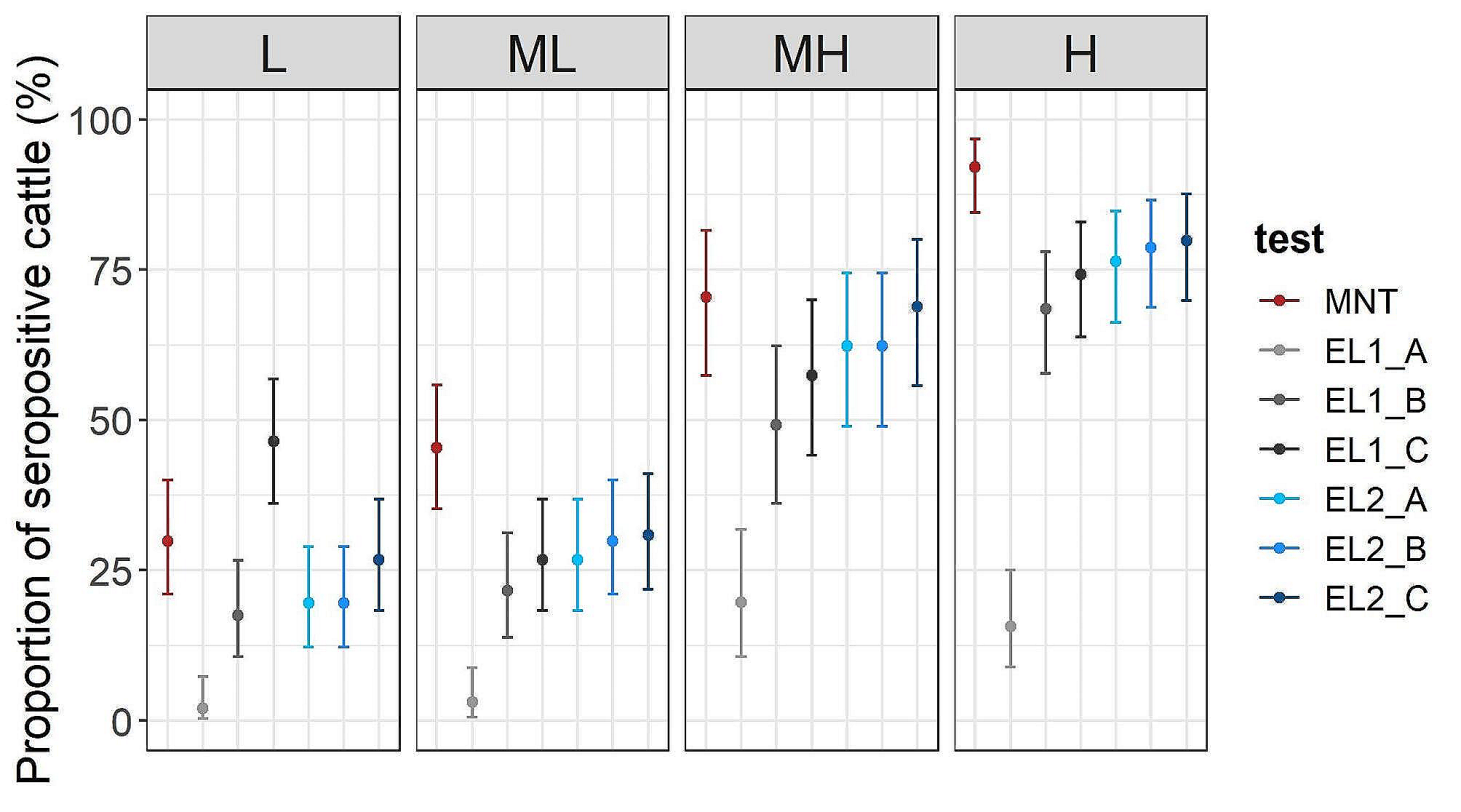
Proportion of seropositive cattle for each grid cell using MNT and cELISA kits with different thresholds to determine the number of positive sera. EL1: ID Screen® cELISA, EL2: Immunozym cELISA, A: cELISA-positive and -doubtful serum according to the threshold recommended by the manufacturer confirmed to be MNT-positive (scenario A); B: cELISA-positive serum according to the adjusted threshold weighting sensitivity and specificity and confirmed MNT-positive (scenario B), C: cELISA-positive serum according to the adjusted threshold weighting sensitivity and specificity (scenario C)

We then estimated seroprevalence according to several scenarii according to the test and the screening strategy used, i.e. serological tests and threshold values chosen for cELISA kits (Fig. 5). We used sera tested with cELISA and positive and doubtful sera tested in MNT using cELISA threshold recommended by the manufacturer (scenario A), or using our adjusted threshold weighting sensitivity and the specificity equally (scenario B). In addition we used positive cELISA results with our adjusted threshold for sera directly tested with cELISA (scenario C). For Immunozym cELISA, overall seroprevalence was underestimated compared with MNT by 9–14% regardless of the screening method (from 43.9% with scenario A to 49.1% with scenario C). On the contrary, the overall estimated seroprevalence was significantly underestimated when using positive and doubtful results based on ID Screen cELISA® (threshold not adjusted) confirmed by MNT (scenario A) compared with the MNT test (9.0% and 57.6%, respectively), and reached 50.0% when using only cELISA positive results (scenario C). Furthermore, the underestimation of cELISA-based seroprevalence per cell varied according to the observed MNT-based seroprevalence in MNT per cell. Underestimation was relatively higher in L and ML cells (MNT-based seroprevalence < 50%) than in MH and H cells and (MNT-based seroprevalence > 50%).

## Discussion

We conducted the first TBEV seroprevalence survey in France, focusing on the historical TBEV endemic area in France, to evaluate exposure of ruminants to TBEV — for use as a baseline for comparison in future studies — and to assess the performance of cELISA tests to determine the best screening method. Here, we highlighted a high and unsuspected level of exposure with 30 to 90% of cattle testing seropositive by MNT locally. We observed that the sensitivity of commercial veterinary cELISA kits was low (< 78%), even after adjusting the positive threshold to the species tested (cattle), and varied according to local virus circulation. Therefore, considering only doubtful and positive cELISA results that have been confirmed by MNT as seropositive for TBEV can underestimate TBEV seroprevalence.

In the French endemic area, we observed very high exposure of cattle to TBEV (57.6%), varying locally from 29.9 to 92.1% seropositive cattle per cell. These values are higher than the seroprevalence typically reported in domestic ruminants in European countries, even in those with higher levels of TBE incidence [40]. The three studies conducted on cattle in highly endemic countries (for review, see ref [40]) reported seroprevalence rates ranging from 2.4% in Lithuania (*n* = 423) to 26.5% in northern Hungary (*n* = 260, varying from 21.2 to 31.4%). Observed seroprevalence in small ruminants in highly endemic countries are also lower than ours were, with a maximum of 32.5% positive sheep (*n* = 310) and of 19.7% positive goats (*n* = 418) found in the Czech Republic [41]. Substantial differences among ruminant herds have nevertheless been observed with seroprevalence as high as 88% in a herd of cows in Norway [25] and 83% in a herd of goats in Switzerland [26]. The different screening strategies (MNT, ELISA, hemagglutination inhibition assay (HAI), doubtful and/or positive cELISA or HAI results subsequently tested using MNT, etc.) might explain to some level the differences in seroprevalence. Indeed, contrary to most studies in which animal sera are tested using ELISA with confirmatory MNT, we directly tested all sera using MNT, which proved to induce higher seroprevalence in our study and hence may have a higher sensitivity than current ELISA kits for detecting TBEV antibodies in animal sera. However, we still observed high seroprevalence using cELISA followed by MNT for confirmation (scenario A and B) except for ID Screen®-scenario A. In any case, our results indicate that the virus circulates intensively in the Vosges Mountains. In this mountainous part of France, cattle graze outside in the mountains and large wooden areas and are likely to be regularly exposed to infected tick bites. In our study, the titre of MNT antibodies varied with the level of seroprevalence. In areas of intense virus circulation, animals are more likely to regularly come into contact with infected ticks, thereby boosting their immune system and raising the titre detected using MNT.

Contrary to expectations considering the supposed long-lived immunity [24], we did not observe an increase in seroprevalence with age. In the high TBEV risk area in north-eastern Hungary, Šikutová et al. (2009) observed a difference of seroprevalence between animals of less than three years old compared with the other age classes of ]3–5], ]5–8] and 8 + year-old cattle, with young animals being less exposed to TBEV [30]. We only sampled animals older than 21 months, but Šikutová et al. (2009) did not report the minimum age of the animals sampled. Therefore, the difference observed between both studies may be due to a difference in the proportion of cattle younger than 20 months. Those young cattle were inevitably less exposed to potential tick bites and may be raised differently than the older age classes (e.g. kept inside stables). However, we did observe a slight, but non-significant difference in the MNT titre distribution according to age, when taking into account the effect of cell seroprevalence. This involved only young cattle less than three years old, having a lower proportion of high MNT titres than the other categories. The majority of these animals may have been less often exposed to the virus than older classes, with little or no immune system boosts, thereby leading to lower seroneutralising antibody titres. The absence of strong variations in seroprevalence and seroneutralising antibody titres according to age class perhaps suggests that natural immunity against TBEV in cattle may not last a lifetime in the absence of new contact with the virus (see also [21]). Alternatively, effect of age may not be apparent here due to variation TBEV exposure levels of cattle that come from different herds.

To date, there are no published data on the performance of commercially available screening methods for the detection of TBEV antibodies in cattle and in considering various epidemiological contexts. We observed low relative sensitivity compared to MNT especially for ID Screen® cELISA (15.4% for ID Screen® vs. 75.1% for Immunozym) when using the doubtful thresholds recommended by the respective manufacturers. When we used optimal cutoff weighting sensitivity and specificity equally for cattle sera, ID Screen® cELISA still showed a significantly lower ability to detect positive cattle (Se, 64.5%) than Immunozym cELISA (Se, 78.1%). This low ability to detect TBEV antibodies in different mammal species has already been observed in previous studies [35, 37–39]. In these studies, the sensitivity of Immunozym cELISA was even lower than ours, with a sensitivity of 57% in goats [35], 42% in foxes [38] and 23% in wild boar sera [37], even all considering borderline results as positive. Only one other study found higher sensitivity, testing dog serum (84.8%, considering only Immunozym cELISA positive results) [39]. For ID Screen® cELISA sensitivity, our results were similar to those reported in the Trozzi et al. (2023) study testing wild boar sera [37]. The relative specificity for both tests was very high (> 90%), when using the doubtful manufacturer’s thresholds. In the Immunozym cELISA, the specificity was higher (≥ 98%) in previous studies in goats [35], foxes [38] and dogs [39] compared with our study (90%), except for the study testing wild boar sera in Belgium (88%) [37]. In that study, they found high seropositivity in wild boar for USUV, leading to cross-reactivity results for both cELISA. We found a very high specificity (99%) for ID Screen® cELISA and all samples that presented positive and doubtful results were tested USUV-negative with MNT. This suggests that cattle may have only been weakly exposed to USUV in our study. However, we cannot exclude that USUV or other circulating flaviviruses were responsible of some positive cross-reactions with Immunozym cELISA results as in healthy blood donors in Switzerland [42]. Although ID Screen® cELISA has been recommended as a pan-flavivirus diagnostic tool in horses [34] and is used to detect TBEV in goats and sheep [43], it was not as effective as expected to detect TBEV antibodies in cattle sera. This test was initially developed to detect antibodies directed against the envelope (E) protein of WNV in avian and horse populations. According to the phylogenetic tree of flaviviruses based on E protein amino acid diversity, WNV and the TBEV serocomplex share only 61% of identity [44], which may explain why the sensitivity of ID Screen® cELISA is low. On the other hand, the Immunozym cELISA kit was specifically developed for TBEV. The test strips are coated with inactivated whole TBEV viral particles (strain Neudoerfl) and a G protein peroxidase conjugate is used as a marker of the bound anti-TBEV antibodies. Sensitivity and specificity on cattle sera remained nevertheless lower than the values stated in Immunozym’s cELISA manual to detect antibodies against TBEV in human sera: 97% and 99%, respectively. The degree of agreement with MNT (ƙ) was less than perfect, being only substantial (0.68). The relatively poor performance of this cELISA on animal sera may be explained by the use of human serum as the internal positive control and calibrators. A modified version using cattle — or at least small ruminant — sera as positive control and calibrators may improve the performance of the test. One study used a modified human version of Immunozym (Immunozym FSME IgM kit, Progen Biotechnik GmbH) to detect IgM and IgG for veterinary purposes after adjusting the threshold [35]. The sensitivity and the specificity were higher (89% and 95%, respectively) than the Immunozym cELISA kit we used in detecting positive goat samples. However, this kit is no longer available for veterinary use [37].

Furthermore, we found that the ability of both cELISA kits to detect positive samples was even lower when the level of neutralising antibodies was below 1:40. Trozzi et al. (2023) observed the same low ability of both cELISA kits to detect TBEV antibodies in wild boar sera [37]. As a result, the relative sensitivity of both cELISA kits was influenced by seroprevalence in our study, because the frequency of low neutralising antibody titre varied with seroprevalence. In low endemicity areas or for juveniles, many animals have been little exposed to TBEV and thus have low antibody titres. IgG antibody levels may decrease over time and more rapidly than neutralising antibodies, reaching the limit of detection of the cELISA kit used. In addition to the difference linked to the species tested, this relationship may also partly explain why there is a large variation in the estimations of sensitivity of the Immunozym cELISA kit in different studies [35, 37–39]. For example, in the Trozzi et al. (2023) study [37], relative sensitivity was only of 23% (in contrast to 75% in our study) in wild boar sera and was calculated from 51% of serum having a neutralising antibody titre lower than 1:40 (17% in our study).

Our study confirmed that using cELISAs for screening may lead to a great underestimation of TBEV seroprevalence in domestic ruminants, especially for ID Screen® cELISA, without a positive threshold adjusted to the species to improve sensitivity and minimize the number of false negative results. Using our sample set, seroprevalence in cattle would have been estimated around 9% with ID Screen® cELISA and 44% with Immunozym cELISA with the recommended thresholds confirmed by MNT, instead of 58% directly using MNT, the gold standard test. The underestimation of cattle exposure is expected to be even higher in low-endemic areas, where the sensitivity of these cELISAs is lower. As a result, there is a risk of not detecting potential new foci using these cELISA tests as screening methods. To partially alleviate the risk of false-negative results without directly testing all samples using MNT, the positive cutoff can be adjusted to increase sensitivity, but it will necessarily be to the detriment of specificity, even in the absence of exposure to USUV. This strategy will require confirmatory MNT testing on a higher number of sera to identify the virus causing the seroconversion. In large serological surveys, an alternative would be to determine the threshold corresponding to a specificity of at least 95% to minimize the cost and time required for the MNT tests.

To compare the performance of two commercially available veterinary cELISA kits to MNT for the detection of TBEV antibodies in cattle, we used 84 cattle sera from western France as a control to evaluate specificity. Surprisingly, three samples used as negative controls for detecting TBEV antibodies were positive in MNT with low titres (≤ 1:40) in western France purportedly free from TBEV. Moreover, these three sera were negative for USUV, another flavivirus that circulates in France [36]. These cattle had never lived in any other region, and have therefore been infected locally. In the absence of RNA screening for the virus, it is impossible to determine which virus from the TBEV serocomplex (non distinguishable by MNT) was responsible for the infection. Indeed, other TBEV serocomplex viruses affecting small ruminants have already been detected in northern Spain, Spanish sheep encephalitis virus, Spanish goat encephalitis virus [2, 45]. The presence of TBEV in western and south-western France might also be a possibility. For example, TBEV was detected in questing ticks in the United Kingdom in 2019 [46].

## Conclusions

We observed that cattle of any age were highly exposed to TBEV in the centre of the French historical endemic region, at an unexpected level considering the human incidence level in France. Such high exposure may be very local and restricted to the Vosges Mountains, where environmental conditions are highly suitable for virus circulation and transmission to cattle, i.e. grazing in open wooded pastures. Seroprevalence and antibody titres were relatively similar across age classes, suggesting that antibodies are detectable for only a few years. It would be interesting to further explore cattle exposure to TBEV as proxy of exposure of domestic ungulates in another high-risk region, e.g. eastern France, to better assess TBEV distribution and potential risk of food-borne transmission. However, the choice of the screening strategy and serological tests strongly influence the estimation of seroprevalence. Here, we showed that two commercial cELISA kits commonly used for detecting TBEV antibodies in animal sera have relatively low sensitivity compared with MNT in cattle sera, and that sensitivity varies according to the local intensity of virus circulation. This reduced test effectiveness has the potential to greatly underestimate seroprevalence. It is therefore important to recognize that the serological screening strategy will affect the estimation of seroprevalence, and the strategy should be chosen according to the aim of the study.

## Materials and methods

### Samples and sampling strategy

The present study constituted a component of a larger investigation designed to assess the degree of exposure of cattle to TBEV in five departments of north-eastern France (results not published). These samples were obtained from the serum libraries of veterinary laboratories from two French departments (Haut-Rhin and Vosges, NUTS 3 administrative level), as part as the mandatory national brucellosis and infectious bovine rhinotracheitis prevention campaign conducted in 2017–2019. In order to have a probability of detecting a seroprevalence of 3% with 95% confidence within each 10 × 10 km² cell given a test sensibility of 95% [47], the aim was to collect 100 sera of cattle which were in the same farm for at least the last three years before sampling, as recorded in the French cattle identification tracing system (*Base de Données Nationale d’Identification*, BDNI). The number of sera did not reach the desired objective for various reasons, due to a higher than expected number of cattle changing farms in the three years prior to sampling. The initial screening test employed was the ID Screen® cELISA kit. Doubtful and positive results (adjusted doubtful threshold, sample (S) to negative control (N): S/N ≤ 70%) were tested using MNT for TBEV and USUV. Based on these results, four cells (Fig. 1) were selected to represent various levels of viral circulation intensity varying from 15 to 70%. A total of 344 bovine serum samples were tested. The cells were named according to the level of TBEV circulation: low circulation (“L” cell), medium low circulation (“ML” cell), medium high circulation (“MH” cell) and high circulation (“H” cell) with 97, 97, 61 and 89 sera tested, respectively.

In addition, to obtain negative sera to calibrate the threshold values for cELISA tests, the National Reference Laboratory for Bluetongue (ANSES, Maisons-Alfort) provided 84 blood serum samples from 15 presumed TBEV-free departments in western France collected in 2019 and 2020 as part of the bluetongue surveillance programme (Fig. 3).

### Age of cattle

Date of birth was obtained from the national livestock database (BDNI). The exact sampling date was not available; therefore, we calculated approximate animal age based on a sampling date arbitrarily set to the middle of the sampling campaign (1 February). Cattle ages varied from 21 months to nearly 21 years. We defined four age classes: [1–3], ]3–5], ]5–8] and 8 + years old, which corresponded to 81, 92, 78 and 83 sera tested, respectively.

### Serological tests

All serum samples were analysed using ID Screen® and Immunozym cELISAs, which are both two-step cELISAs. ID Screen® cELISA uses plates pre-coated with West Nile virus (WNV) recombinant antigens and a monoclonal anti-E antibody conjugated to horseradish peroxidase (HRP) as markers for the bound anti-TBE-IgG antibodies. In Immunozym cELISA, test strips are coated with inactivated TBEV, strain Neudoerfl, with a protein G peroxidase conjugate as the marker. The tests were carried out according to the manufacturer’s instructions. Optical density (OD) was measured at 450 nm for both tests. The result for ID Screen® cELISA was the competition percentage (S/N) calculated using the sample (S) OD and dividing it by the negative (N) control OD then multiplying by 100 (S/N * 100). To calculate the antibody concentration of each sample in Vienna units (VIEU/mL) with Immunozym cELISA, we fitted the OD to a non-linear regression model built with the five calibrators (known concentration in antibodies) provided by the manufacturer.

Sera were also analysed using micro-neutralisation tests (MNTs, strain Hypr, GenBank ID U39292.1) to confirm the presence of specific neutralising antibodies against TBEV [48]. Because cELISA results can cross-react with antibodies of other flaviviruses, we also performed MNTs for the detection of specific neutralising antibodies against USUV (strain Italy 2012, 206795-3/2012, GenBank ID KX816653.1, provided by Davide Lelli, IZSLER, Brescia, Italy). USUV is another flavivirus that circulates in France, especially in the eastern part of the country in 2016 [49]. Only doubtful and positive samples detected with ID Screen® cELISA, using the threshold provided by the manufacturers, were screened for USUV antibodies using MNT because ID Screen® cELISA performs better than Immunozym cELISA to detect USUV antibodies [37]. A sample was considered positive if antibodies to TBEV and USUV were detected at a serum dilution of at least 1:20 and 1:10, respectively. Three titre groups were designated according to the level of neutralising antibodies against TBEV in serum: titres ranging from 1:20 to 1:40 (low titre), titres from 1:80 to 1:160 (medium titre) and titres greater than 1:320 (high titre).

### Epidemiological characteristics of TBEV circulation in the endemic area

We estimated the seroprevalence of neutralising antibodies for each cell and for each age class from the ratio of MNT positive samples to the total number of samples, with the exact binomial confidence intervals of 95% (95% CI). To test if the serological status of cattle varied with age, we modelled it as a function of age class using binomial generalized linear mixed models (GLMM) with the function glmer in the lme4 package in R® software. Cell and herd were included as nested random factors to account for potential aggregation of virus circulation.

We then assessed the distribution of MNT titres regarding the intensity of viral circulation and age categories from the proportion of each group of MNT titres among positive samples per age class and per cell. We used a multinomial logistic regression model (function multinom in the nnet package in R® software) to test the distribution of MNT titre categories according to age class and intensity of virus circulation per cell. For this analysis, the intensity of virus circulation was studied by including the “cell” or by grouping cells into two categories according to their seroprevalence: “less than 50%” or “greater than 50%”.

### cELISA performance compared with MNT

To compare the performance of cELISA with MNT, we used the ROCR package (implemented in R® software) to draw the receiver operating characteristic (ROC) curve, which plots the false positive rate (1-specificity) on the x-axis and the true positive rate (sensitivity) on the y-axis. We compared performance using (i) the threshold provided by the manufacturer (considering borderline samples as positive) and (ii) using an optimal positive threshold for bovine species for each cELISA. This optimal cutoff was the value of S/N (ID Screen® cELISA) or concentration (Immunozym cELISA) that weighted both sensitivity and specificity equally. This threshold was then used to determine the serological status of the sample analysed by cELISA (number of positive samples with each cELISA). The area under the curve (AUC) from each ROC curve was also calculated [50].

Using MNT results as the reference assay, we calculated relative sensitivity (Se) to evaluate the ability of the cELISA test to detect an MNT-positive animal and relative specificity (Sp) to determine the ability of the test to detect an MNT-negative animal. A highly sensitive test minimizes the number of false negatives. A highly specific test limits the number of false positives. Se and Sp were calculated as follows:

Se = (TP / (TP + FN)) * 100

Sp = (TN / (TN + FP)) * 100

With TP, FN, TN, FP being the number of true positive, false negative, true negative and false positive sera according to MNT results. The kappa coefficient (ƙ) [51] was also calculated to measure the level of agreement between each cELISA test and MNT, as follows:

ƙ = (P - P_c_) / (1 – P_c_)

where P is the relative agreement among tests, calculated as P = (TP + TN) / n.

and P_c_ the hypothetical probability of chance of agreement: P_c_ = [(TP + FN) * (TP + FP) + (TN + FP) * (TN + FN)] / n² with n = FP + FN + TP + TN.

Based on this value, agreement was deemed poor (ƙ ≤ 0), slight (0.01 ≤ ƙ ≤ 0.20), fair (0.21 ≤ ƙ ≤0.40), moderate (0.41 ≤ ƙ ≤0.60), substantial (0.61 ≤ ƙ ≤ 0.80) or almost perfect (0.81 ≤ ƙ ≤ 1).

We then assessed the performance of cELISA kits to detect positive samples according to the titre in MNT and quantified the correlation between the cELISA value (S/N or VIEU/mL) and titre in MNT using a Spearman’s rank correlation test.

Finally, we evaluated the impact of the serological screening method to estimate the seroprevalence for each cell. Generally, ELISA are used as a first screening method and then positive and doubtful results are tested in MNT [19, 30, 41, 52]. Therefore, we considered the following strategies, using positive MNT results for sera directly tested in MNT (scenario MNT), sera tested with cELISA and positive and doubtful sera tested in MNT using cELISA threshold recommended by the manufacturer (scenario A), or using our adjusted threshold weighting sensitivity and the specificity equally (scenario B); (ii) using positive cELISA results with our adjusted threshold for sera directly tested with cELISA (scenario C).

All data were processed using R® software version 4.1.1® (R Development Core Team 2021-08-10).

## Data Availability

The datasets of the current study are available from the corresponding author upon reasonable request.

## Abbreviations

BDNI: Base de Données Nationale d’Identification
ECDC: European Centre for Disease Prevention and Control
ELISA: Enzyme-liked immunabsorbent assay
IBR: Infectious bovine rhinotracheitis
MNT: Micro virus neutralisation test
TBEV: Tick-borne encephalitis virus
